# Supplementary material for: Effect of Age on Heart Rate Variability in Patients with Mitral Valve Prolapse: An Observational Study
Source: J Clin Med. 2022 Dec 25;12(1):165. doi: 10.3390/jcm12010165 (PMC9820965; doi:10.3390/jcm12010165)
Supplement: Supplementary file 1 [file jcm-12-00165-s001.zip › jcm-1915666-supplementary.pdf]

**Table S1. The full results of multivariable quantile regression for the association between MVP and HRV parameters**

| HRV parameters/ predictors | Regression coefficient (95% CI) | P value |
|---|---|---|
| SDNN, ms | | |
| MVP | 3.6 (−8.6, 15.8) | 0.560 |
| Age, year | -0.37 (-0.69, -0.05) | 0.024 |
| Male | 9.5 (-3.6, 22.5) | 0.154 |
| Smoking | −6.3 (−24.1, 11.6) | 0.490 |
| RMSSD, ms | | |
| MVP | −1.5 (−7.3, 4.2) | 0.602 |
| Age, year | -0.17 (−0.32, -0.02) | 0.025 |
| Male | −1.8 (−8.0, 4.3) | 0.555 |
| Smoking | 6.9 (−1.4, 15.3) | 0.104 |
| NN50 | | |
| MVP | 38.3 (−2494.6, 2571.2) | 0.976 |
| Age, year | −156.4 (−223.3, -89.6) | <0.001 |
| Male | −1336.3 (−4041.3, 1368.6) | 0.331 |
| Smoking | 3816.1 (116.9, 7515.3) | 0.043 |
| PNN50, % | | |
| MVP | 0.35 (−2.49, 3.19) | 0.810 |
| Age, year | −0.13 (-0.21, −0.06) | 0.001 |
| Male | −1.4 (−4.4, 1.7) | 0.369 |
| Smoking | 4.2 (0.0, 8.3) | 0.049 |
| RR interval mean, ms2 | | |
| MVP | 5.6 (−36.2, 47.4) | 0.792 |
| Age, year | 2.56 (1.46, 3.67) | <0.001 |
| Male | −7.9 (-52.5, 36.8) | 0.729 |
| Smoking | 6.9 (−54.1, 68.0) | 0.823 |
| VLF, ms2 | | |
| MVP | 246.1 (−749.4, 1241.5) | 0.626 |
| Age, year | 25.4 (−0.7, 51.5) | 0.056 |
| Male | −473.3 (−1530.6, 583.9) | 0.378 |
| Smoking | 365.0 (−1090.8, 1820.9) | 0.621 |
| LF, ms2 | | |
| MVP | −81.5 (−1128.5, 965.4) | 0.878 |
| Age, year | 37.7 (10.2, 65.1) | 0.007 |
| Male | −330.0 (−1442.0, 781.9) | 0.559 |
| Smoking | 239.1 (-1292.0, 1770.3) | 0.758 |
| HF, ms2 | | |
| MVP | −79.1 (−713.7, 555.5) | 0.806 |
| Age, year | 13.3 (-3.3, 29.9) | 0.117 |
| Male | −297.6 (−971.6, 376.4) | 0.385 |
| Smoking | 253.6 (-674.5, 1181.7) | 0.590 |
| Total, ms2 | | |

| | | |
|---|---|---|
| MVP | −229.5 (−2821.7, 2362.7) | 0.861 |
| Age, year | 69.5 (1.6, 137.4) | 0.045 |
| Male | −797.5 (−3550.6, 1955.7) | 0.568 |
| Smoking | −268.5 (−4059.5, 3522.5) | 0.889 |
| LF/HF ratio | | |
| MVP | −0.10 (−0.51, 0.32) | 0.653 |
| Age, year | −0.01 (−0.02, 0.01) | 0.367 |
| Male | −0.02 (−0.46, 0.42) | 0.929 |
| Smoking | 0.02 (−0.59, 0.62) | 0.961 |

CI, confidence interval.
